# GbyE: an integrated tool for genome widely association study and genome selection based on genetic by environmental interaction

**DOI:** 10.1186/s12864-024-10310-5

**Published:** 2024-04-19

**Authors:** Xinrui Liu, Mingxiu Wang, Jie Qin, Yaxin Liu, Shikai Wang, Shiyu Wu, Ming Zhang, Jincheng Zhong, Jiabo Wang

**Affiliations:** 1 Key Laboratory of Qinghai-Tibetan Plateau Animal Genetic Resource Reservation and Utilization, Sichuan Province and Ministry of Education, Southwest Minzu University, Chengdu, 6110041 China; 2 Nanchong Academy of Agricultural Sciences, Nanchong, 637000 China

**Keywords:** Genome-widely association study, Genomic selection, Software, R, GbyE

## Abstract

**Background:**

The growth and development of organism were dependent on the effect of genetic, environment, and their interaction. In recent decades, lots of candidate additive genetic markers and genes had been detected by using genome-widely association study (GWAS). However, restricted to computing power and practical tool, the interactive effect of markers and genes were not revealed clearly. And utilization of these interactive markers is difficult in the breeding and prediction, such as genome selection (GS).

**Results:**

Through the Power-FDR curve, the GbyE algorithm can detect more significant genetic loci at different levels of genetic correlation and heritability, especially at low heritability levels. The additive effect of GbyE exhibits high significance on certain chromosomes, while the interactive effect detects more significant sites on other chromosomes, which were not detected in the first two parts. In prediction accuracy testing, in most cases of heritability and genetic correlation, the majority of prediction accuracy of GbyE is significantly higher than that of the mean method, regardless of whether the rrBLUP model or BGLR model is used for statistics. The GbyE algorithm improves the prediction accuracy of the three Bayesian models BRR, BayesA, and BayesLASSO using information from genetic by environmental interaction (G × E) and increases the prediction accuracy by 9.4%, 9.1%, and 11%, respectively, relative to the Mean value method. The GbyE algorithm is significantly superior to the mean method in the absence of a single environment, regardless of the combination of heritability and genetic correlation, especially in the case of high genetic correlation and heritability.

**Conclusions:**

Therefore, this study constructed a new genotype design model program (GbyE) for GWAS and GS using Kronecker product. which was able to clearly estimate the additive and interactive effects separately. The results showed that GbyE can provide higher statistical power for the GWAS and more prediction accuracy of the GS models. In addition, GbyE gives varying degrees of improvement of prediction accuracy in three Bayesian models (BRR, BayesA, and BayesCpi). Whatever the phenotype were missed in the single environment or multiple environments, the GbyE also makes better prediction for inference population set. This study helps us understand the interactive relationship between genomic and environment in the complex traits. The GbyE source code is available at the GitHub website (https://github.com/liu-xinrui/GbyE).

**Supplementary Information:**

The online version contains supplementary material available at 10.1186/s12864-024-10310-5.

## Background

Genetic by environmental interaction (G × E) is crucial of explaining individual traits and has gained increasing attention in research. It refers to the influence of genetic factors on susceptibility to environmental factors. In-depth study of G × E contributes to a deeper understanding of the relationship between individual growth, living environment and phenotypes. Genetic factors play a role in most human diseases at the molecular or cellular level, but environmental factors also contribute significantly. Researchers aim to uncover the mechanisms behind complex diseases and quantitative traits by investigating the interactions between organisms and their environment. Common, complex, or rare human diseases are often considered as outcomes resulting from the interplay of genes, environmental factors, and their interactions. Analyzing the joint effects of genes and the environment can provide valuable insights into the underlying pathway mechanisms of diseases. For instance, researchers have successfully identified potential loci associated with asthma risk through G × E interactions [1], and have explored predisposing factors for challenging-to-treat diseases like cancer [2, 3], rhinitis [4], and depression [5].

However, two main methods are currently being used by breeders in agricultural production to increase crop yields and livestock productivity [6]. The first is to develop varieties with relatively low G × E effect to ensure stable production performance in different environments. The second is to use information from different environments to improve the statistical power of genome-wide association study (GWAS) to reveal potential loci of complex traits. The first method requires long-term commitment, while the second method clearly has faster returns. In GWAS, the use of multiple environments or phenotypes for association studies has become increasingly important. This not only improves the statistical power of environmental susceptibility traits[7], but also allows to detect signaling loci for G × E. There are significant challenges when using multiple environments or phenotypes for GWAS, mainly because most diseases and quantitative traits have numerous associated loci with minimal impact [8], and thus it is impossible to determine the effect size regulated by environment in these loci. The current detection strategy for G × E is based on complex statistical model, often requiring the use of a large number of samples to detect important signals [9, 10]. In GS, breeders can use whole genome marker data to identify and select target strains in the early stages of animal and plant production [11–13]. Initially, GS models, similar to GWAS models, could only analyze a single environment or phenotype [14]. To improve the predictive accuracy of the models, higher marker densities are often required, allowing the proportion of genetic variation explained by these markers to be increased, indirectly obtaining higher predictive accuracy. It is worth mentioning that the consideration of G × E and multiple phenotypes in GS models [15] has been widely studied in different plant and animal breeding [16]. GS models that allow G × E have been developed [17] and most of them have modeled and interpreted G × E using structured covariates [18]. In these studies, most of the GS models provided more predictive accuracy when combined with G × E compared to single environment (or phenotype) analysis. Hence, there is need to develop models that leverage G × E information for GWAS and GS studies.

This study developed a novel genotype-by-environment method based on R, termed GbyE, which leverages the interaction among multiple environments or phenotypes to enhance the association study and prediction performance of environmental susceptibility traits. The method enables the identification of mutation sites that exhibit G × E interactions in specific environments. To evaluate the performance of the method, simulation experiments were conducted using a dataset comprising 282 corn samples. Importantly, this method can be seamlessly integrated into any GWAS and GS analysis.

## Materials and methods

### Support packages

The development purpose of GbyE is to apply it to GWAS and GS research, therefore it uses the genome association and prediction integrated tool (GAPIT) [19], Bayesian Generalized Linear Regression (BGLR) [20], and Ridge Regression Best Linear Unbiased Prediction (rrBLUP) [21]package as support packages, where GbyE only provides conversion of interactive formats and file generation. In order to simplify the operation of the GbyE function package, the basic calculation package is attached to this package to support the operation of GbyE, including four function packages GbyE.Simulation.R (Dual environment phenotype simulation based on heritability, genetic correlation, and QTL quantity), GbyE.Calculate.R (For numerical genotype and phenotype data, this package can be used to process interactive genotype files of GbyE), GbyE.Power.FDR.R (Calculate the statistical power and false discovery rate (FDR) of GWAS), and GbyE.Comparison.Pvalue.R (GbyE generates redundant calculations in GWAS calculations, and SNP effect loci with minimal *p*-values can be filtered by this package).

### Samples and sequencing data

In this study, a small volume of data was used for software simulation analysis, which is widely used in testing tasks of software such as GAPIT, TASSEL, and rMPV. The demonstration data comes from 282 inbred lines of maize, including 4 phenotypic data. In any case, there are no missing phenotypes in these data, and this dataset can be obtained from the website of GAPIT (https://zzlab.net/GAPIT/index.html, accessed on May 1, 2022). Among them, our phenotype data was simulated using a self-made R simulation function, and the Mean and GbyE phenotype files were calculated. Convert this format to HapMap format using PLINK v1.09 and scripts written by oneself.

### Simulated traits

Phenotype simulation was performed by modifying the GAPIT.Phenotype.Simulation function in the GAPIT. Based on the input parameter NQTN, the random selected markers’ genotype from whole genome were used to simulate genetic effect in the simulated trait. The genotype effects of these selected QTNs were randomly sampled from a multivariate normal distribution, the correlation value between these normal distribution was used to define the genetic relationship between each environments. The additive heritability (${{\text{h}}}_{{\text{g}}}^{2}$) was used to scale the relationship between additive genetic variance and phenotype variance. The simulated phenotype conditions in this paper are set as follows: 1) The three levels of ${{\text{h}}}_{{\text{g}}}^{2}$ were set at 0.8, 0.5, and 0.2, representing high (${{\text{h}}}_{{\text{h}}}^{2}$), median (${{\text{h}}}_{{\text{m}}}^{2}$) and low (${{\text{h}}}_{{\text{l}}}^{2}$) heritability; 2) Genetic correlation were set three levels 0.8, 0.5, 0.2 representing high (${{\text{R}}}_{{\text{h}}}$), medium (${{\text{R}}}_{{\text{m}}}$) and low (${{\text{R}}}_{{\text{l}}}$) genetic correlation; 3) 20 pre-set effect loci of QTL. The phenotype values in each environment were simulated together following above parameters.

### Genetic by environment interaction model

**Fig. 1:**
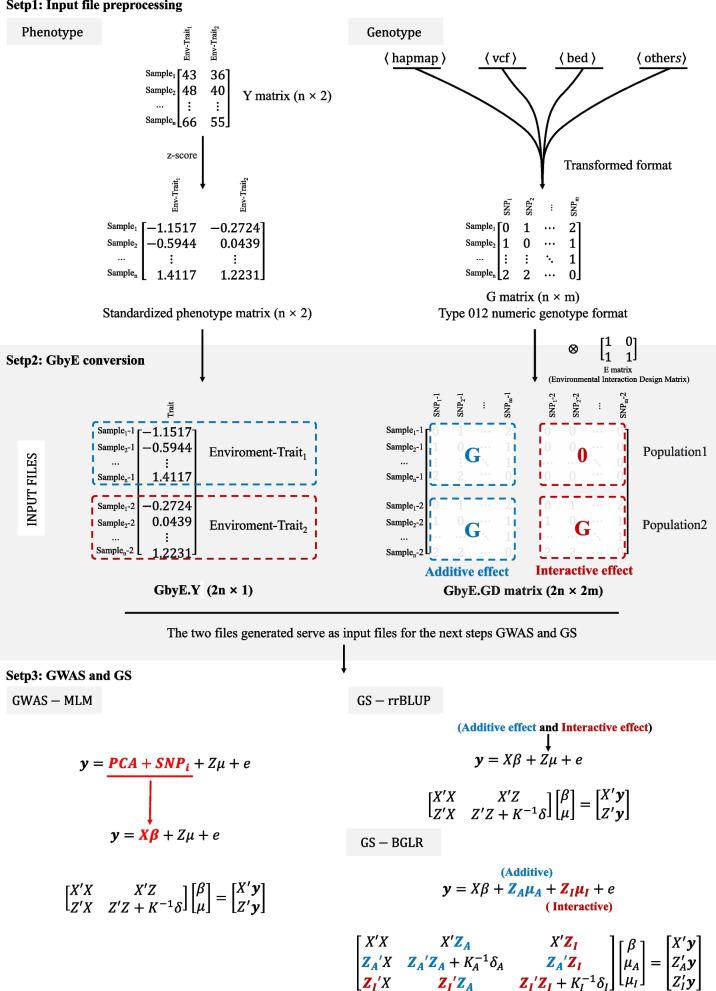
The workflow pipeline of GbyE. The GbyE contains three main steps. (Step 1) Preprocessing of phenotype and genotype data,. The phenotype values in each environment was normalized respectively. Meanwhile, all genotype from HapMap, VCF, BED, and other types were converted to numeric genotype; (Step 2) Generate GbyE phenotype and interactive genotype matrix through the transformation of GbyE. In GbyE.GD matrix, the blue characters indicate additive effect, and red ones indicate interactive effect; (Step 3) The MLM and rrBLUP and BGLR were used to perform GWAS and GS

The pipeline analysis process of GbyE includes three steps: data preprocessing, production converted, Association analysis. Normalize the phenotype data matrix Y of the dual environment and perform GbyE conversion to generate phenotype data in GbyE.Y format. The genotype data format, such as hapmap, vcf, bed and other formats firstly need to be converted into numerical genotype format (homozygotes were coded as 0 or 2, heterozygotes were coded as 1) using software or scripts such as GAPIT, PLINK, etc. The environment (E) matrix is environment index matrix. The G (n × m) originally of genotype matrix was converted as GbyE.GD(2n × 2 m) $$\left[\begin{array}{cc}G& 0\\ G& G\end{array}\right]$$ during the Kronecker product, and the Y vector (n × 1) was also converted as the GbyE.Y vector (2n × 1) after normalization. The duplicated data format indicated different environments, genetic effect, and populations. The genomic data we used in the analysis was still retained the whole genome information. The first column of E is the additive effect, which was the average genetic effect among environments. The others columns of E are the interactive effect, which should be less one column than the number of environments. Because it need to avoid the linear dependent in the model. In the GbyE algorithm, we coded the first environment as background as default, that means the genotype in the first environment are 0, the others are 1. Then the Kronecker product of G and environment index matrix was named as GbyE.GD. The interactive effect part of the GbyE.GD matrix in the GWAS and GS were the relative values based on the first environment (Fig. 1). The GbyE environmental interaction matrix can be easily obtained by constructing the interaction matrix E (e.g., Eq. 1) such that the genotype matrix G is Kronecker-product with the design interaction matrix E (e.g., Eq. 2), in which $$\left[\begin{array}{c}G\\ G\end{array}\right]$$ matrix is defined as additive effect and $$\left[\begin{array}{c}0\\ G\end{array}\right]$$ matrix is defined as interactive effect. $$\left[\begin{array}{cc}G& 0\\ G& G\end{array}\right]$$ matrix is called gene by environment interaction matrix, hereinafter referred to as the GbyE matrix. The phenotype file (GbyE.Y) and genotype file (GbyE.GD) after transformation by GbyE will be inputted into the GWAS and GS models and computed as standard phenotype and genotype files.

1 $${\text{E}}=\left[\begin{array}{cc}1& 0\\ 1& 1\end{array}\right]$$

2 $${\text{G}}\otimes {\text{E}}=\left[\begin{array}{cc}{\text{G}}& 0\\ {\text{G}}& {\text{G}}\end{array}\right]$$

where G is the matrix of whole genotype and E is the design matrix for exploring interactive effects. GbyE mainly uses the Kronecker product of the genetic matrix (G) and the environmental matrix (E) as the genotype for subsequent GWAS as a way to distinguish between additive and interactive effects.

### Association analysis model

The mixed linear model (MLM) of GAPIT is used as the basic model for GWAS analysis, and the principal component analysis (PCA) parameter is set to 3. Then the *p*-values of detection results are sorted and their power and FDR values are calculated. General expression of MLM (Fig. 1):

3 $$Y=PCA+{SNP}_{i}+Z\mu +e$$

where Y is the vector of phenotypic measures (2n × 1); PCA and SNP_i_ were defined as fixed effects, with a size of (2n × 2 m); Z is the incidence matrix of random effects; μ is the random effect vector, which follows the normal distribution μ ~ N(0, ${\delta }_{G}^{2}$﻿K) with mean vector of 0 and variance covariance matrix of ${\delta }_{G}^{2}$﻿K, where the ${\delta }_{G}^{2}$ is the total genetic variance including additive variance and interactive variance, the K is the kinship matrix built with all genotype including additive genotype and interactive genotype; e is a random error vector, and its elements need not be independent and identically distributed, e ~ N(0,${\delta }_{e}^{2}$ I), where the ${\delta }_{e}^{2}$ is the residual and environment variance, the I is the design matrix.

### Detectivity of GWAS

In the GWAS results, the list of markers following the order of P-values was used to evaluate detectivity of GWAS methods. When all simulated QTNs were detected, the power of the GWAS method was considered as 1 (100%). From the list of markers, following increasing of the criterion of real QTN, the power values will be increasing. The FDR indicates the rate between the wrong criterion of real QTNs and the number of all un-QTNs. The mean of 100 cycles was used to consider as the reference value for statistical power comparison. Here, we used a commonly used method in GWAS research with multiple traits or environmental phenotypes as a comparison[22]. This method obtains the mean of phenotypic values under different conditions as the phenotypic values for GWAS analysis, called the Mean value method, Compare the calculation results of GbyE with the additive and interactive effects of the mean method to evaluate the detection power of the GbyE strategy. Through the comprehensive analysis of these evaluation indicators, we aim to comprehensively evaluate the statistical power of the GbyE strategy in GWAS and provide a reference for future optimization research.

Among them, the formulae for calculating Power and FDR are as follows:

4 $${\text{Power}}=\frac{{\sum }_{{\text{i}}}^{{{\text{m}}}_{{\text{r}}}}{{\text{n}}}_{{\text{i}}}}{{{\text{m}}}_{{\text{r}}}}$$

where ${{\text{n}}}_{{\text{i}}}$ indicates whether the i-th detection is true, true is 1, false is 0; ${{\text{m}}}_{{\text{r}}}$ is the total number of all true QTLs in the sample size; the maximum value of Power is 1.

5 $${\text{FDR}}=\frac{{\sum }_{{\text{i}}}^{{{\text{M}}}_{{\text{f}}}}{{\text{N}}}_{{\text{i}}}}{{{\text{M}}}_{{\text{f}}}}$$

where ${{\text{N}}}_{{\text{i}}}$ represents the i-th true value detected in the pseudogene, true is 1, false is 0. and cumulative calculation; ${{\text{M}}}_{{\text{f}}}$ is the number of all labeled un-QTNs in the total samples; the maximum value of FDR is 1.

### Genomic prediction

To comparison the prediction accuracy of different GS models using GbyE, we performed rrBLUP, Bayesian methods using R packages. All phenotype of reference population and genotype of all population were used to train the model and predict genomic estimated breeding value (gEBV) of all individuals. The correlation between real phenotypes and gEBV of inference population was considered as prediction accuracy. fivefold cross-validation and 100 times repeats was performed to avoid over prediction and reduce bias. In order to distinguish the additive and interactive effects in GbyE, we designed two lists of additive and interactive effects in the "ETA" of BGLR, and put the additive and interactive effects into the model as two kinships for random objects. However, it was not possible to load the gene effects of the two lists in rrBLUP, so the additive and interactive genotypes together were used to calculate whole genetic kinship in rrBLUP (Fig. 1). Relevant parameters in BGLR are set as follows: 1) model set to "RRB"; 2) nIter is set to "12000"; 3) burnIn is set to "10000". The results of the above operations are averaged over 100 cycles. We also validated the GbyE method using four other Bayesian methods (BayesA, BayesB, BayesCpi, and Bayesian LASSO) in addition to RRB in BGLR.

### Partial missing phentoype in the prediction

In this study, we artificially missed phenotype values in the single and double environments in the whole population from 281 inbred maize datasets. In the missing single environment case, the inference set in the cross-validation was selected from whole population, and each individual in the inference were only missed phenotypes in the one environment. The phenotype in the other environment was kept. The genotypes were always kept. In the case of missing double environments, both phenotypes and genotypes of environment 1 and environment 2 are missing, and the model can only predict phenotypic values in the two missing environments through the effects of other markers. In addition, the data were standardized and unstandardized to assess whether standardization had an effect on the estimation of the model. This experiment was tested using the "ML" method in rrBLUP to ensure the efficiency of the model.

## Results

### GWAS statistical power of models at different heritabilities and genetic correlations

**Fig. 2:**
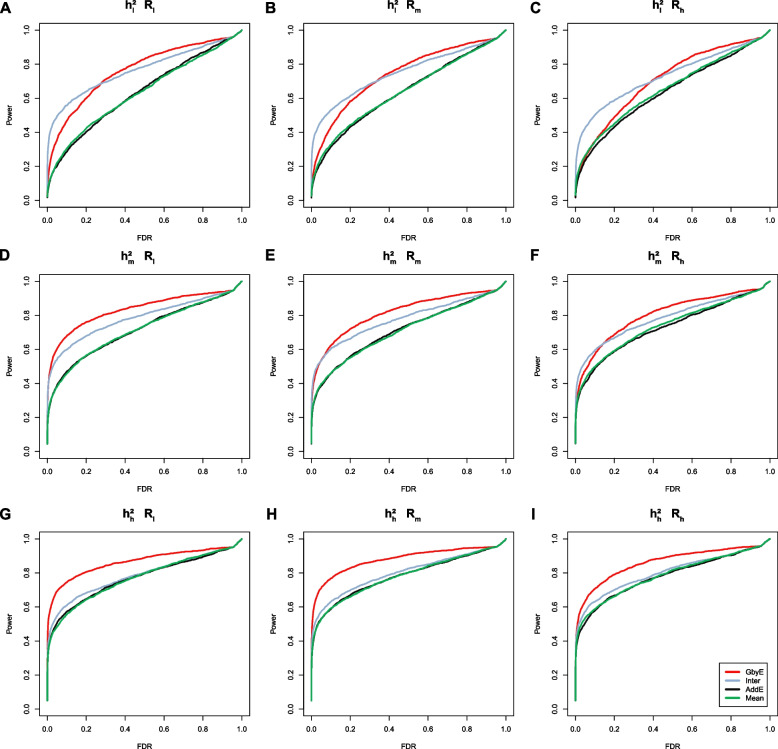
The power-FDR testing in simulated traits. Comparing the efficacy of the GbyE algorithm with the conventional mean method in terms of detection power and FDR. From left to right, the three levels of genetic correlation are indicated in order of low, medium and high. From top to bottom, the three levels of heritability, low, medium and high, are indicated in order. (1) Inter: Interactive section extracted from GbyE; (2) AddE: Additive section extracted from GbyE; (3) ${{\text{h}}}_{{\text{l}}}^{2}$, ${{\text{h}}}_{{\text{m}}}^{2}$, ${{\text{h}}}_{{\text{g}}}^{2}$: Low, medium, high heritability; (4) ${{\text{R}}}_{{\text{l}}}$, ${{\text{R}}}_{{\text{m}}}$, ${{\text{R}}}_{{\text{l}}}$: where R stands for genetic correlation, represents three levels of low, medium and high

Power-FDR plots were used to demonstrate the detection efficiency of GbyE at three genetic correlation and three genetic power levels, with a total of nine different scenarios simulated (from left to right for high and low genetic correlation and from top to bottom for high and low genetic power). In order to distinguish whether the effect of improving the detection ability of genome-wide association analysis in GbyE is an additive effect or an effect of environmental interactions, we plotted their Power-FDR curves separately and added the traditional Mean method for comparative analysis. As shown in Fig. 2, GbyE algorithm can detect more statistically significant genetic loci with lower FDR under any genetic background. However, in the combination with low heritability (Fig. 2A, B, C), the interactive effect detected more real loci than GbyE under low FDR, but with the continued increase of FDR, GbyE detected more real loci than other groups. Under the combination with high heritability, all groups have high statistical power at low FDR, but with the increase of FDR, the statistical effect of GbyE gradually highlights. From the analysis of heritability combinations at all levels, the effect of heritability on interactive effect is not obvious, but GbyE always maintains the highest statistical power. The average detection power of GWAS in GbyE can be increased by about 20%, and with the decrease of genetic correlation, the effect of GbyE gradually highlights, indicating that the G × E plays a role.

### Resolution of additive and interactive effect

**Fig. 3:**
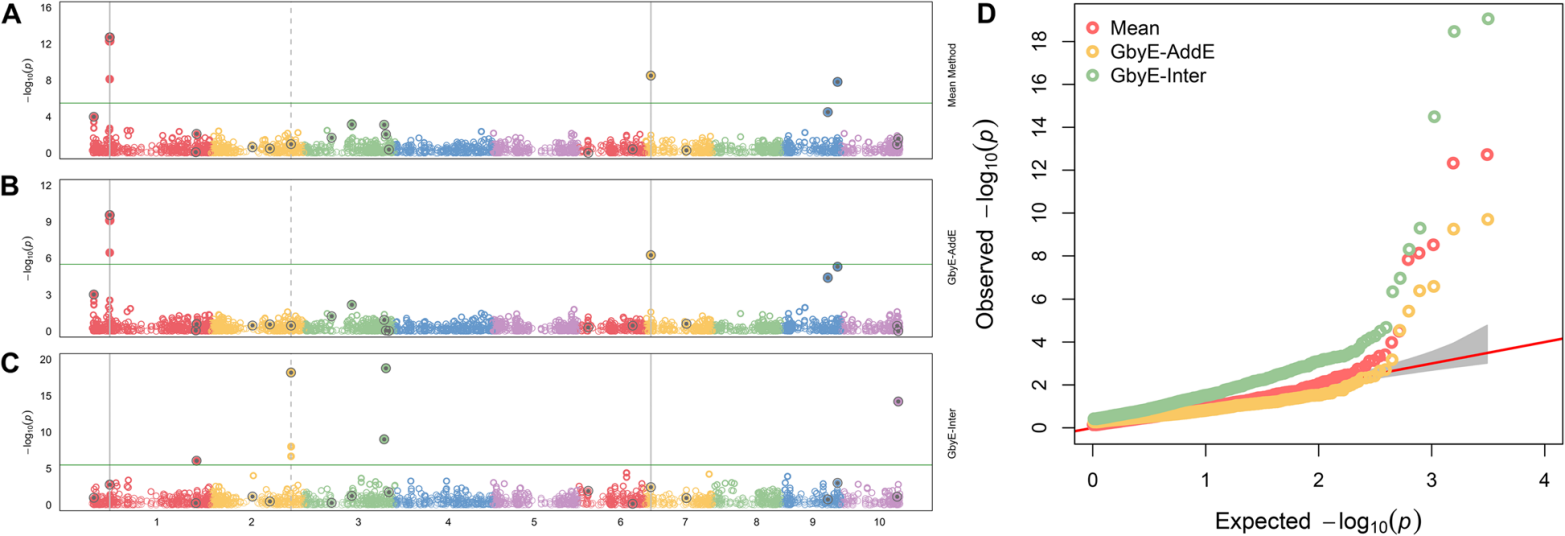
Manhattan statistical comparison plot. Manhattan comparison plots of mean (**A**), additive (**B**) and gene-environment interactive sections (**C**) at a heritability of 0.9 and genetic correlation of 0.1. Different colors are used in the diagram to distinguish between different chromosomes (X-axis). Loci with reinforcing circles and centroids are set up as real QTN loci. Consecutive loci found in both parts are shown as id lines, and loci found separately in the reciprocal effect only are shown as dashed lines. Parallel horizontal lines indicate significance thresholds (*p*-value < 3.23 × 10^–6^). **D** Quantile–quantile plots of simulated phenotypes for demo data from genome-wide association studies. x-axis indicates expected values of log *p*-values and y-axis is observed values of log *p*-values. The diagonal coefficients in red are 1. GbyE-inter is the interactive section in GbyE; GbyE-AddE is the additive section in GbyE

The output results of GbyE could be understood as resolution of additive and interactive genetic effect. Hence, we created a combined Manhattan plots with Mean result from MLM, additive, and interactive results from GbyE. As shown in Fig. 3, true marker loci were detected on chromosomes 1, 6 and 9 in Mean, and the same loci were detected on chromosomes 1 and 6 for the additive result in GbyE (the common loci detected jointly by the two results were marked as solid gray lines in the figure). All known pseudo QTNs were labeled with gray dots in the circle. Total 20 pseudo QTNs were simulated in such trait (The heritability is set to 0.9, and the genetic correlation is set to 0.1). Although the additive section in GbyE did not catch the locus on chromosome 9 yet (those p-values of markers did not show above the significance threshold (p-value < 3.23 × 10^–6^)), it has shown high significance relative to other markers of the same chromosome. In the reciprocal effect of GbyE, we detected more significant loci on chromosomes 1, 2, 3 and 10, and these loci were not detected in either of the two previous sections. An integrate QQ plot (Fig. 3D) shows that the overall statistical power of the additive section in Mean and GbyE are close, nevertheless, the interactive section in the GbyE provided a bit of inflation.

### Genomic selection in assumption codistribution

**Fig. 4:**
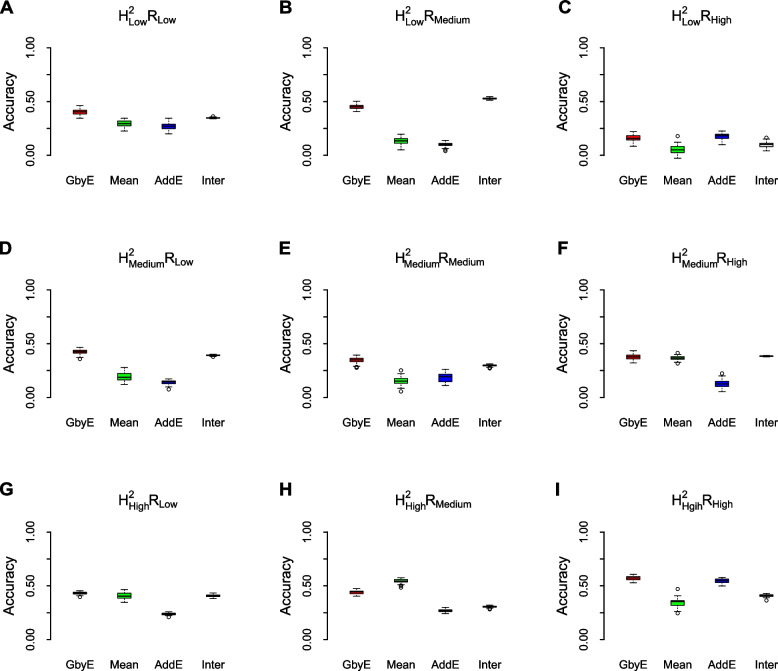
Box-plot of model prediction accuracy. The prediction accuracy (pearson's correlation coefficient) of the GbyE algorithm was compared with the tradition al Mean value method in a simulation experiment of genomic selection under the rrBLUP operating environment. The effect of different levels of heritability and genetic correlation on the prediction accuracy of genomic selection was simulated in this experiment. Each row from top to bottom represents low heritability (${{\text{h}}}_{{\text{l}}}^{2}$), medium heritability (${{\text{h}}}_{{\text{m}}}^{2}$) and high heritability (${{\text{h}}}_{{\text{h}}}^{2}$), respectively; each column from left to right represents low genetic correlation (${{\text{R}}}_{{\text{l}}}$), medium genetic correlation (${{\text{R}}}_{{\text{m}}}$) and high genetic correlation (${{\text{R}}}_{{\text{h}}}$), respectively; The X-axis shows the different test methods and effects, and the Y-axis shows the prediction accuracy

The prediction accuracy of GbyE was significantly higher than the Mean value method by model statistics of rrBLUP in most cases of heritability and genetic correlation (Fig. 4). The prediction accuracy of the additive effect was close to that of Mean value method, which was consistent with the situation under the low hereditary. The prediction accuracy of interactive sections in GbyE remains at the same level as in GbyE, and interactive section plays an important role in the model. We observed that in ${{\text{h}}}_{{\text{l}}}^{2}{{\text{R}}}_{{\text{h}}}$ (Fig. 4C), ${{\text{h}}}_{{\text{m}}}^{2}{{\text{R}}}_{{\text{h}}}$ (Fig. 4F), ${{\text{h}}}_{{\text{h}}}^{2}{{\text{R}}}_{{\text{l}}}$ (Fig. 4G), the prediction accuracy of GbyE was slightly higher than the Mean value method, but there was no significant difference overall. In addition, we only observed that the prediction accuracy of GbyE was slightly lower than the Mean value method in ${{\text{h}}}_{{\text{h}}}^{2}{{\text{R}}}_{{\text{l}}}$ (Fig. 4H), but there was still no significant difference between GbyE and Mean value methods. Under the combination of low heritability and genetic correlation, the prediction accuracy of Mean value method and additive effect model remained at a similar level. However, with the continuous increase of heritability and genetic correlation, the difference in prediction accuracy between the two gradually increases. In summary, the GbyE algorithm can improve the accuracy of GS by capturing information on multiple environment or trait effects under the rrBLUP model.

### Genomic selection in assumption un-codistribution

The overall performance of GbyE under the 'BRR' statistical model based on the BGLR package remained consistent with rrBLUP, maintaining high predictive accuracy in most cases of heritability and genetic relatedness (Fig. S1). However, when the heritability is set to low and medium, the difference between the prediction accuracy of GbyE algorithm and Mean value method gradually decreases with the continuous increase of genetic correlation, and there is no statistically significant difference between the two. The prediction accuracy of the model by GbyE in ${{\text{h}}}_{{\text{h}}}^{2}{{\text{R}}}_{{\text{l}}}$ (Fig. S1G) and ${{\text{h}}}_{{\text{h}}}^{2}{{\text{R}}}_{{\text{h}}}$ (Fig. S1I) is significantly higher than that by Mean value method when the heritability is set to be high. On the contrary, when the genetic correlation is set to medium, there is no significant difference between GbyE and Mean value method in improving the prediction accuracy of the model, and the overall mean of GbyE is lower than Mean. When GbyE has relatively high heritability and low genetic correlation, its prediction accuracy is significantly higher than the mean method, such as ${{\text{h}}}_{{\text{m}}}^{2}{{\text{R}}}_{{\text{l}}}$ (Fig. S1D), ${{\text{h}}}_{{\text{h}}}^{2}{{\text{R}}}_{{\text{l}}}$ (Fig. S1G), and ${{\text{h}}}_{{\text{h}}}^{2}{{\text{R}}}_{{\text{m}}}$ (Fig. S1H). Therefore, GbyE is more suitable for situations with high heritability and low genetic correlation.

### Adaptability of Bayesian models

**Fig. 5:**
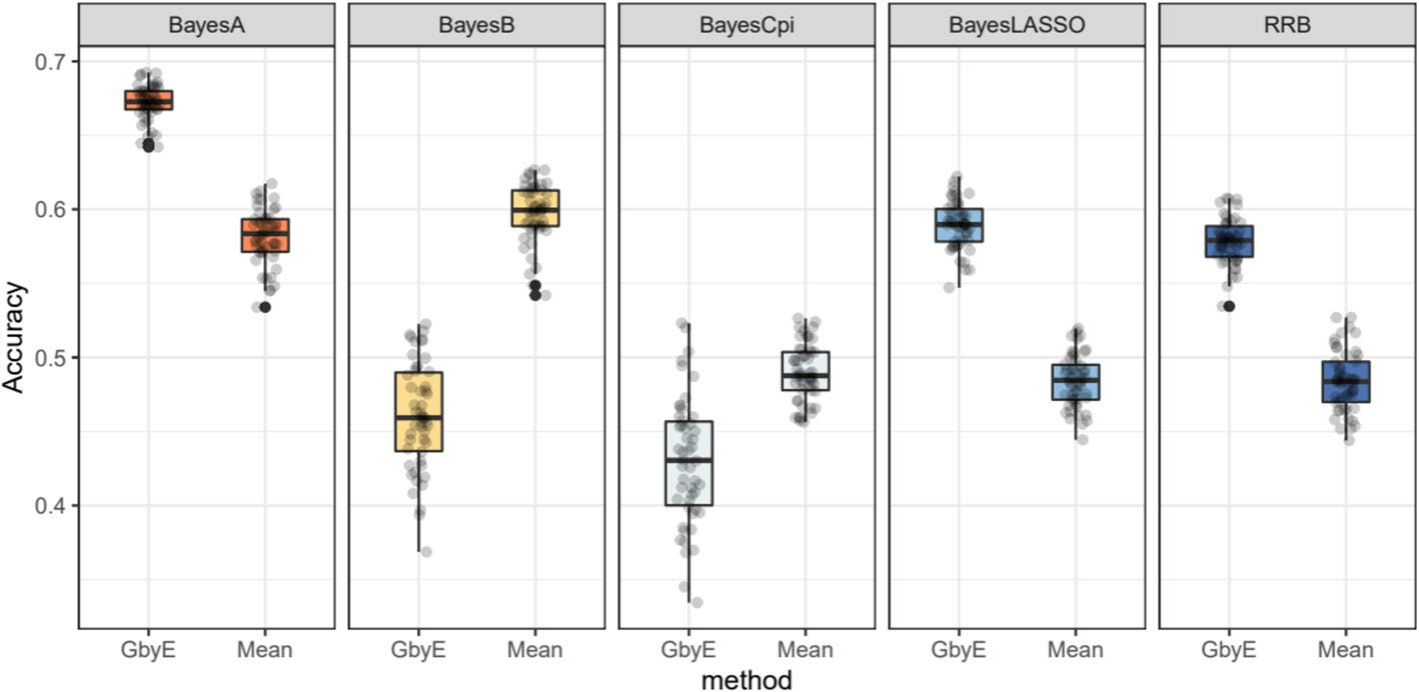
Relative prediction accuracy histogram for different Bayesian models. The X-axis is the Bayesian approach based on BGLR, and the Y-axis is the relative prediction accuracy. Where we normalize the prediction accuracy of Mean (the prediction accuracy is all adjusted to 1); the prediction accuracy of GbyE is the increase or decrease value relative to Mean in the same group of models

Next, we tested a more complex Bayesian model. The GbyE algorithm and Mean value method were combined with five Bayesian algorithms in BGLR for GS analysis, and the computing R script was used for phenotypic simulation test, where heritability and genetic correlation were both set to 0.5. The results indicate that among the three Bayesian models of RRB, BayesA, and BayesLASSO, the predictive accuracy of GbyE is significantly higher than that of Mean value method (Fig. 5). In contrast, under the Bayesian models of BayesB and BayesCpi, the prediction accuracy of GbyE is lower than that of the Mean value method. The GbyE algorithm improves the prediction accuracy of the three Bayesian models BRR, BayesA, and BayesLASSO using information from G × E and increases the prediction accuracy by 9.4%, 9.1%, and 11%, respectively, relative to the Mean value method. However, the predictive accuracy of the BayesB model decreased by 11.3%, while the BayescCpi model decreased by 6%.

### Impact of all and partial environmental missing

**Fig. 6:**
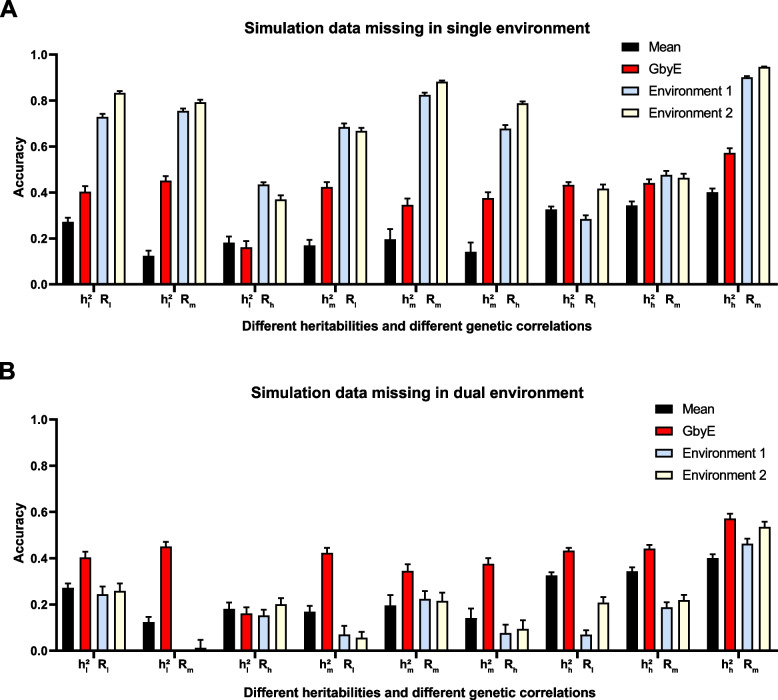
Prediction accuracy of simulated data in single and dual environment absence. The prediction effect of GbyE was divided into two parts, environment 1 and environment 2, to compare the prediction accuracy of GbyE when predicting these two parts separately. This includes simulations with missing phenotypes and genotypes in environment 1 only (**A**) and simulations with missing in both environments (**B**). The horizontal coordinates of the graph indicate the different combinations of heritabilities and genetic correlations of the simulations

We tested missing the environmental by using simulated data. In the case of the simulated data, we simulated a total of nine situations with different heritability and genetic correlations (Fig. 6) and conducted tests on single and dual environment missing. The improvement in prediction accuracy by the GbyE algorithm was found to be significantly higher than the Mean value method in single environment deletion, regardless of the combination of heritability and genetic correlation. In the case of ${{\text{h}}}_{{\text{h}}}^{2}{{\text{R}}}_{{\text{h}}}$, the prediction accuracy of GbyE is higher than 0.5, which is the highest value among all simulated combinations. When GbyE estimates the phenotypic values of Environment 1 and Environment 2 separately, its predictive accuracy seems too accurate. On the other hand, when the phenotypic values of both environments are missing on the same genotype, the predictive accuracy of GbyE does not show a significant decrease, and even maintains accuracy comparable to that of a single environment missing. However, when GbyE estimates Environment 1 and Environment 2 separately, the prediction accuracy significantly decreases compared to when a single environment is missing, and the prediction accuracy of Environment 1 and Environment 2 in ${{\text{h}}}_{{\text{l}}}^{2}{{\text{R}}}_{{\text{m}}}$ is extremely low (Fig. 6B). In addition, the prediction accuracy of GbyE is lower than Mean values only in ${{\text{h}}}_{{\text{l}}}^{2}{{\text{R}}}_{{\text{h}}}$, whether it is missing in a single or dual environment.

## Discussion

The phenotype of organisms is usually controlled by multiple factors, mainly genetic [23] and environmental factors [24], and their interactive factors. The phenotype of quantitative traits is often influenced by these three factors [25, 26]. However, based on the computing limitation and lack of special tool, the interactive effect always was ignored in most GWAS and GS research, and it is difficult to distinguish additive and interactive effects. The rate between all additive genetic variance and phenotype variance was named as narrow sense heritability. The accuracy square of prediction of additive GS model is considered that can not surpass narrow sense heritability. In this study, the additive effects in GbyE are essentially equivalent to the detectability of traditional models, the key advantage of GbyE is the interactive section. More significant markers with interactive effects were detected. Detecting two genetic effects (additive and interactive sections) in GWAS and GS is a boost to computational complexity, while obtaining genotypes for genetic interactions by Kronecker product is an efficient means. This allows the estimation of additive and interactive genetic effects separately during the analysis, and ultimately the estimated genetic effects for each GbyE genotype (including additive and interactive genetic effect markers) are placed in a t-distribution for *p*-value calculation, and the significance of each genotype is considered by multiple testing. The GbyE also expanded the estimated heritability as generalized heritability which could be explained as the rate between total genetics variance and phenotype variance.

The genetic correlation among traits in multiple environments is the major immanent cause of GbyE. When the genetic correlation level is high, then additive genetic effects will play primary impact in the total genetic effect, and interactive genetic effects with different traits or environments are often at lower levels [27]. Therefore, the statistical power of the GbyE algorithm did not improve significantly compared with the traditional method (Mean value) when simulating high levels of genetic correlation. On the contrary, in the case of low levels of genetic correlation, the genetic variance of additive effects is relatively low and the genetic variance of interactive effects is major. At this time, GbyE utilizes multiple environments or traits to highlight the statistical power. Since the GbyE algorithm obtains additive, environmental, and interactive information by encoding numerical genotypes, it only increases the volume of SNP data and can be applied to any traditional GWAS association statistical model. However, this may slightly increase the correlation operation time of the GWAS model, but compared to other multi environment or trait models [28, 29], GbyE only needs to perform a complete traditional GWAS once to obtain the results.

In GS, rrBLUP algorithm is a linear mixed model-based prediction method that assumes all markers provide genetic effects and their values following a normal distribution [30]. In contrast, the BGLR model is a linear mixed model, which assumes that gene effects are randomly drawn from a multivariate normal distribution and genotype effects are randomly drawn from a multivariate Gaussian process, which takes into account potential pleiotropy and polygenic effects and allows inferring the effects of single gene while estimating genomic values [31]. The algorithm typically uses Markov Chain Monte Carlo methods for estimation of the ratio between genetic variances and residual variances [32, 33]. The model has been able to take into account more biological features and complexity, and therefore the overall improvement of the GbyE algorithm under BGLR is smaller than Mean method. In addition, the length of the Markov chain set on the BGLR package is often above 20,000 to obtain stable parameters and to undergo longer iterations to make the chain stable [34]. GbyE is effective in improving the statistical power of the model under most Bayesian statistical models. In the case of the phenotypes we simulated, more iterations cannot be provided for the BayesB and BayesCpi models because of the limitation of computation time, which causes low prediction accuracy. It is worth noting that the prediction accuracy of BayesCpi may also be influenced by the number of QTLs [35], and the prediction accuracy of BayesB is often related to the distribution of different allele frequencies (from rare to common variants) at random loci [36].

The overall statistical power of GbyE was significantly higher in missing single environment than in missing double environment, because in the case of missing single environment, GbyE can take full advantage of the information from the phenotype in the second environment. And the correlation between two environments can also affect the detectability of the GbyE algorithm in different ways. On the one hand, a high correlation between two environments can improve the predictive accuracy of the GbyE algorithm by using the information from one environment to predict the breeding values in the other environment, even if there is only few relationship with that environment [37, 38]. On the other hand, when two environments are extremely uncorrelated, GbyE algorithm trained in one environment may not export well to another environment, which may lead to a decrease in prediction accuracy [39]. In the testing, we found that when the GbyE algorithm uses a GS model trained in one environment and tested in another environment, the high correlation between environments may result to the model capturing similarities between environments unrelated to G × E information [40]. However, when estimating the breeding values for each environment separately, GbyE still made effective predictions using the genotypes in that environment and maintained high prediction accuracy. As expected, the additive effect calculates the average genetic effect between environments, and its predictive effect does not differ much from the mean method. The interactive effect, however, has one less column than the number of environments, and it calculates the relative values between environments, a component that has a direct impact on the predictive effect. The correlation between the two environments may have both positive and negative effects on the detectability of the GbyE, so it is important to carefully consider the relationship between the two environments in subsequent in development and testing.

A key advantage of the GbyE algorithm is that it can be applied to almost all current genome-wide association and prediction. However, the focus of GbyE is still on estimating additive and interactive effects separately, so that it is easy to determine which portion of the is playing a role in the computational estimation.. The GbyE algorithm may have implications for the design of future GS studies. For example, the model could be used to identify the best environments or traits to include in GS studies in order to maximize prediction accuracy. It is particularly important to test the model on large datasets with different genetic backgrounds and environmental conditions to ensure that it can accurately predict genome-wide effects in a variety of contexts.

## Conclusions

GbyE can simulate the effects of gene-environment interactions by building genotype files for multiple environments or multiple traits, normalizing the effects of multiple environments and multiple traits on marker effects. It also enables higher statistical power and prediction accuracy for GWAS and GS. The additive and interactive effects of genes under genetic roles could be revealed clearly, which makes it possible to utilize environmental information to improve the statistical power and prediction accuracy of traditional models, thus helping us to better understand the interactions between genes and the environment.

### Supplementary Information

**Supplementary Material 1.**

## Data Availability

The GbyE source code, demo script, and demo data are freely available on the GitHub website (https://github.com/liu-xinrui/GbyE).

## Abbreviations

GWAS: Genome-widely association study
GS: Genome selection
G × E: Genetic by environmental interaction
GAPIT: Genome association and prediction integrated tool
MLM: Mixed linear model
BGLR: Bayesian generalized linear regression
rrBLUP: Ridge regression best linear unbiased prediction
FDR: False discovery rate
PCA: Principal component analysis
gEBV: Genomic estimated breeding value
