# Supplementary material for: GbyE: an integrated tool for genome widely association study and genome selection based on genetic by environmental interaction
Source: BMC Genomics. 2024 Apr 19;25:386. doi: 10.1186/s12864-024-10310-5 (PMC11027269; doi:10.1186/s12864-024-10310-5)
Supplement: Supplementary file 1 — Supplementary Material 1. [file 12864_2024_10310_MOESM1_ESM.docx]

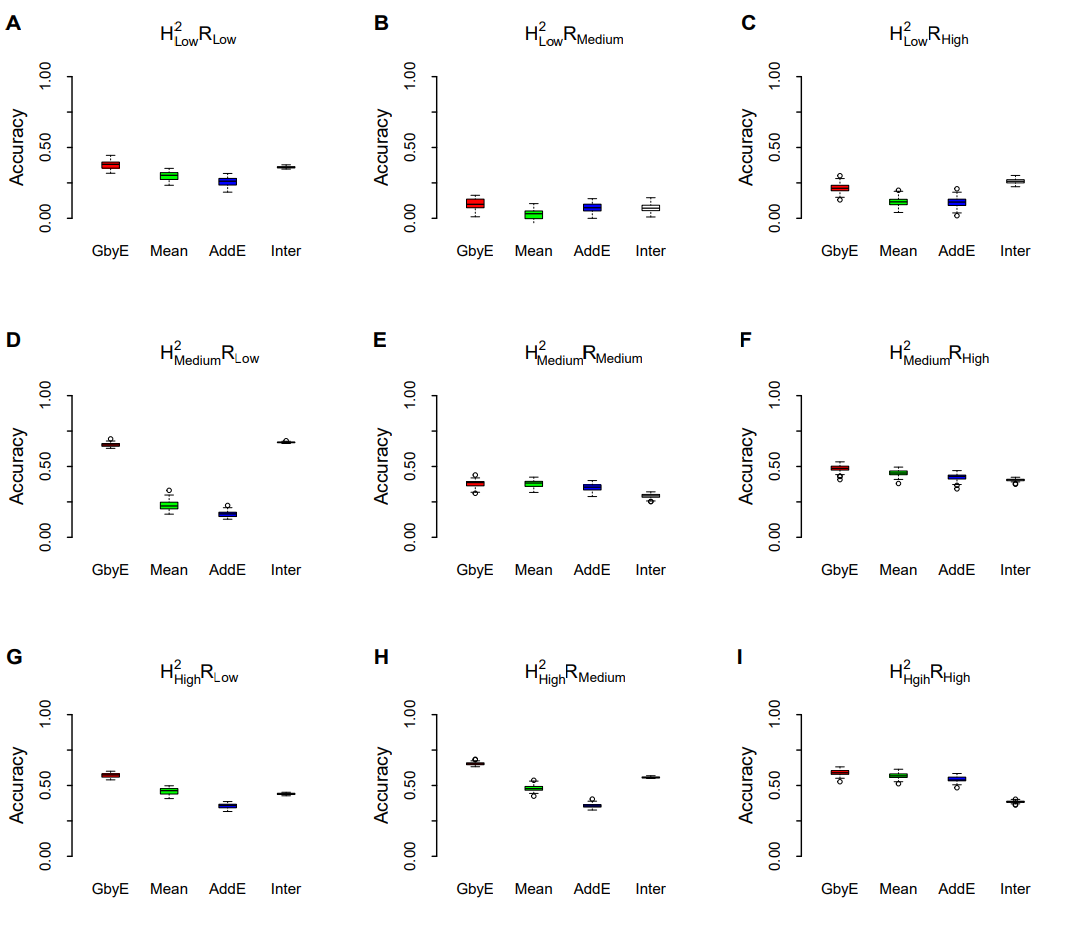


**Supplementary Figure 1.** Box-plot of model prediction accuracy. The prediction accuracy (pearson's correlation coefficient) of the GbyE model was compared with the tradition al Mean value method in a simulation experiment of genomic selection under the rrBLUP operating environment. The effect of different levels of heritability and genetic correlation on the prediction accuracy of genomic selection was simulated in this experiment. Each row from top to bottom represents low heritability ($h_{l}^{2}$), medium heritability ($h_{m}^{2}$) and high heritability ($h_{h}^{2}$), respectively; each column from left to right represents low genetic correlation ($R_{l}$), medium genetic correlation ($R_{m}$) and high genetic correlation ($R_{h}$), respectively; The X-axis shows the different test methods and effects, and the Y-axis shows the prediction accuracy.
